# Effect of transient cerebral ischemia on the expression of receptor for advanced glycation end products in the gerbil hippocampus proper

**DOI:** 10.1186/cc13646

**Published:** 2014-03-17

**Authors:** JH Cho, CW Park, HY Lee, MH Won, JY Lee, DJ Chung

**Affiliations:** 1 Kangwon National University, Chuncheonsi, South Korea

## Introduction

The receptor for advanced glycation end products (RAGE) is a multiligand receptor of the immunoglobulin superfamily that has been implicated in multiple neuronal and inflammatory stress processes. In the present study, we investigated changes in RAGE immunoreactivity and its protein levels in the gerbil hippocampus (CA1 to 3 regions) after 5 minutes of transient global cerebral ischemia.

## Methods

The ischemic hippocampus was stained with cresyl violet (CV), NeuN (a neuron-specific soluble nuclear antigen) antibody and Fluro-Jade B (a marker for neuronal degeneration).

## Results

Five days after ischemia-reperfusion, delayed neuronal death occurred in the stratum pyramidale (SP) of the CA1 region. RAGE immunoreactivity was not detected in any regions of the CA1 to 3 regions of the sham group. RAGE immunoreactivity was detected only in the CA1 region from 3 days post ischemia, and the RAGE immunoreactivity was newly expressed in astrocytes, not in neurons. In addition, the level of RAGE protein was highest at 5 days post ischemia. In brief, both the RAGE immunoreactivity and protein level were distinctively increased in astrocytes in the ischemic CA1 region from 3 days after transient cerebral ischemia.

## Conclusion

These results indicate that the increase of RAGE expression in astrocytes at post ischemia may be related to the ischemia-induced activation of astrocytes in the ischemic CA1 region.

